# Study on creep hardening-damage constitutive model of cemented tailings backfill based on time-harden theory

**DOI:** 10.1371/journal.pone.0319470

**Published:** 2025-03-31

**Authors:** Yufan Feng, Guanghua Sun, Zhiyi Liu

**Affiliations:** 1 College of Mining Engineering, North China University of Science and Technology, Tangshan, Hebei, China; 2 Shen kan Engineering and Technology Corporation, MCC, Shenyang, Liaoning, China; 3 Hebei Key Laboratory of Mining Development and Safety Technology, Tangshan, Hebei, China; 4 Green Intelligent Mining Technology Innovation Center of Hebei Province, Tangshan, Hebei, China; Shandong University of Technology, CHINA

## Abstract

The wide application of the fill mining method is necessary for practicing the development concept of “green mountains are golden mountains” and protecting the surface landscape. In the underground quarry, the cemented tailings backfill (CTB) is mainly subjected to the gravity of the overlying rock layer in the mining area, which will cause creep problems in the long term. In order to protect the long-term stability of the mine surface, to study the creep hardening-damage characteristics of the CTB under the uniaxial action, for the maintenance age of 28d, cement-tailings ratios of 1:6 CTB to carry out uniaxial grading loading creep test, combined with the theory of time hardening Explore the creep process of the CTB in the hardening-damage law and the construction of the creep isomorphic model. The results show that the differences in the creep process of 1:6 cement-tailings ratios filler are caused by the joint existence of hardening and damage effects. In the stage of decelerated creep, creep hardening plays a major role; in the stage of stabilized creep, creep hardening and damage play a joint role; in the stage of accelerated creep, the creep damage effect is dominant; the strength of the CTB is strong and then weakened, and ultimately, the cumulative damage is too large to produce a “Y” type damage. The intrinsic model constructed based on the time-hardening theory better characterizes the creep of the 1:6 cement-tailings ratios of CTB under different stress levels. K and r are the material constants of the CTB, and the parameter K affects the time of decelerated creep during the decelerated creep stage, and the parameter r affects the creep rate of isokinetic creep.

## 1. Introduction

Filling mining method is a way to solve the environmental problems caused by mining activities and waste gangue accumulation at a lower cost [1]. The filling body will carry the gravity of the overlying rock body of the mining area for a long time after filling the mining area, and it will produce creep with the passage of time, and then produce creep damage, and if destabilization damage occurs, it will affect the supporting effect and the safety of the quarry. Therefore, it is of great significance to carry out the research on the creep mechanical properties of the backfill under long-term loading.

Scholars have carried out a large number of studies on the creep characteristics of the backfill and achieved fruitful results [2–4]. Qiu [5] obtained stress-strain curves of fillers with different cement tailings ratios through laboratory mechanical tests. According to the principle of damage mechanics, the damage constitutive model of backfill is established. Meng [6] verified the creep compression characteristics of waste rock backfill through creep compression tests under stepwise loading, and proposed an NMK model to describe its creep compression characteristics. Yin [7] studied the expansion ratio and strength of sulfide cemented paste backfill, and analyzed its hardening process and expansion effect. Based on Weibull distribution density function, the strain equivalent principle and the damage mechanics theory, Tu [8] established a two-stage constitutive model of CTB under uniaxial compression with different solid contents and cement-sand ratios. The theoretical model agrees well with the experimental results. Feng [9] conducted a creep test on cemented gangue backfill column through the step-by-step load method to obtain uniaxial compressive strength and elastic modulus. A rheology-based model that considered the time-hardening of cemented gangue backfill column at early curing age was proposed to predict the time-dependent defor-mation of cemented gangue backfill column starting from early age. Wang [10] studied the creep characteristics of backfill materials under different moisture contents and proposed a fractional Bingham creep damage model, which could better describe the creep process of fill bodies in nonlinear creep stages under different moisture contents. All the above research results are carried out on the single damage or hardening characteristics in the creep process of backfill, without involving the interaction between the two and the influence on the creep mechanical properties when they exist together.

In the process of studying the creep characteristics of rocks and other materials, many scholars have found that the two mechanisms of hardening and damage coexist in the creep process [11,12].CAO studied the strain softening and hardening properties of rock under the influence of pore and volume changes, and established a statistical damage constitutive model for the strain softening and hardening behavior of rock under conventional triaxial compression [13]. By introducing time hardening theory and damage theory, the analytical solution of strengthening and weakening behavior in the whole creep process is obtained [14]. Cai [15] analyzed the hardening-damage mechanism of lean coal creep by carrying out a uniaxial graded loading creep test on lean coal. Chen [16] believes that CTB has an obvious compaction hardening in the initial compaction stage and strain softening at the plastic yield stage. Considering the nonlinear deformation characteristics of CTB, a complete constitutive model of CTB is proposed and verified by FLAC3D.

Liu [17] et al. conducted uniaxial compression creep test on backfill specimens with different cement-sand ratios, and adopted the improved inflection point method of steady state creep rate to determine the long-term strength of backfill. Qi [18,19] et al. believe that the horizontal displacement of rock mass was responsible for the long-term stress development in the backfilled stope. Wang [20] carried out consolidation compression tests were performed for cemented tailings backfill with a cement-sand ratio of 1:10 and a concentration of 70% under confined conditions, the consolidation creep constitutive model of backfill under the high-stress constraint is established. Xu [21] used uniaxial compression test and DIC method, established the corrected damage model of gangue cemented backfill through numerical simulation. Zhang [22] carried out the grading loading creep testing of the backfill under different water pressures, and found that the increase in water pressure degrades the mechanical properties of the backfill, and exacerbates the damage of a backfill sample.

Based on the above results, it is found that most of the studies on the combined effects of hardening and damage in creep process focus on rock-like materials, while few studies on CTB. The creep properties of backfill are similar to rock materials to some extent, but not exactly the same. According to the research results of scholars, there are many creep models to describe the creep deformation of backfill. With the deepening of the research, a large number of parameters have been added to these models to constrain them, which makes the application of creep models inconvenient. Therefore, the uniaxial graded loading creep test of the CTB was carried out to obtain the variation rules of creep rate and elastic modulus during the creep process, analyze the hardening-damage mechanism during the creep process, and introduce the time-hardening function and damage variables to construct an intrinsic model of the creep of the CTB, and then validate the model’s reasonableness. Using fewer creep parameters to describe the creep deformation law of CTB, it is hoped that it can simplify the creep model and provide a new way of thinking for the subsequent research.

## 2. Physical test and result analysis

### 2.1. Uniaxial compression test

The specimens of the CTB were prepared by using the tailing sand and ordinary 32.5# silicate cement in Jidong area, and the standard specimens were prepared according to 75% mass concentration, 1:6 cement-tailings ratios and 2:1 height-diameter ratio. The specimens were maintained in YH-40B standard constant temperature and humidity curing box for 28 days at 20°C and 95% humidity.

A number of filled bodies were divided into two groups, the first group used WHY-600 testing machine, uniaxial compressive strength test, uniaxial compressive strength ($R_{c}$ ) of the CTB, used to determine the grading of the CTB loading stress level, loading rate of 2mm/min, preloading 0.1kN.

**Fig 1:**
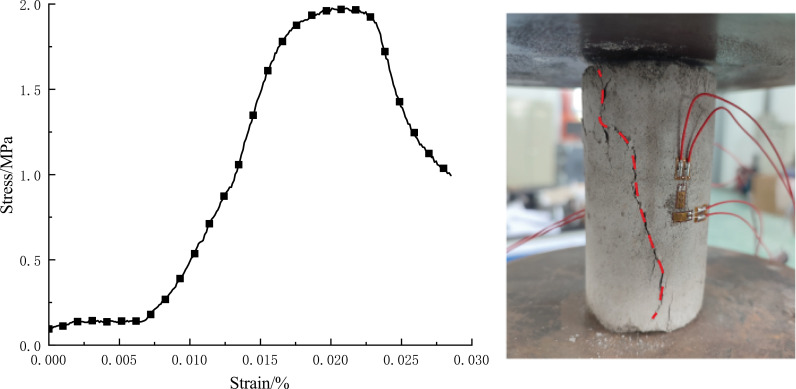
Failure state and full stress-strain curve of CTB under uniaxial compression. (a) Full stress-strain curve of CTB. (b) Failure state of CTB.

The full stress-strain curves and post-damage morphology of the CTB in uniaxial compression tests are shown in Fig 1. During uniaxial compression, with the increase of the stress level, the original pore fissures in the CTB are compacted and closed, the lateral deformation of the specimen is small, and the volume decreases with the increase of the load. The stress-strain curve is nearly straight, and the CTB is in the elastic stage. When the pressure continues to increase, the CTB produces plastic deformation, from the elastic stage to the plastic stage, the cracks of the CTB continue to grow, and the internal stress is released until it is completely destroyed. At the same time, the transverse strain rate of the specimen increases rapidly, and the volume of the CTB changes from compression to expansion. When the stress-strain curve of the CTB reaches the peak strength, the internal structure of the CTB has been destroyed, but basically maintains the overall shape. After the peak strength, the specimen as a whole forms a macroscopic fracture surface, and then slips along the fracture surface, and the specimen’s bearing capacity decreases rapidly, resulting in damage.

### 2.2. Classified creep test

#### 2.2.1. Test equipment.

The second group adopts RBYD-5030 rock creep disturbance tester for creep test with graded loading test. The instrument uses weights or preloads for gravity loading. The instrument can automatically balance and keep the load stable for a long time, without any maintenance, and effectively meet the requirements of stress loading. When the specimen is deformed, the instrument automatically compensates through the load amplification system to ensure that the same stress is always loaded on the specimen, achieving extremely high loading accuracy and loading stability. The maximum axial static load is 30t, the maximum axial displacement range is 10mm, and the accuracy is ± 1%FS. The maximum axial load range is 300kN, the accuracy is ± 1%FS. Strain control 0.05 ~ 1.5mm/min, accuracy ± 1%FS.

#### 2.2.2 Loading method.

There are mainly two types of uniaxial creep loading methods for rock materials: separate loading method and graded loading method. The creep test using the graded loading method utilizes the same instrument to apply load to the same specimen in stages, which saves the testing equipment and specimens, and also reduces the error caused by the differences between specimens. Therefore, the creep test was completed using the graded loading method.70% ～ 85% of the uniaxial compressive strength($R_{c}$) of the specimen as the last level of loading can be obtained reasonable test results, therefore, the specimen uniaxial creep test with 80% $R_{c}$ as the last level of loading, respectively, 20% $R_{c}$, 40% $R_{c}$, 60% $R_{c}$, 80% $R_{c}$ stress level in the same specimen from small to large step-by-step loading, each level of load constant time is 24h [23]. The working face temperature of the tailing sand is about 25°C. In order to be close to the real underground conditions and reduce the influence of different temperatures on the samples, the laboratory temperature is controlled at 25 ± 1°C.

#### 2.2.3. Test procedure.

1) Affix two perpendicular strain gauges in the shape of “⊢” at the center of the specimen to measure its axial and transverse strains, respectively. Attach two sets of strain gauges symmetrically on either side of the specimen. Connect these strain gauges to the static strain gauge, which should then be linked to the tester’s control host.
2) Position the test specimen on the testing machine and place a balancing weight above it to ensure even distribution of the machine’s load onto the specimen. Align the upper and lower surfaces of the specimen with the gaskets, ensuring that the pressure sensor beneath the tester is clear of any debris.
3) Install the displacement meter at the midpoint of the specimen to monitor its radial displacement changes, setting an initial reading of approximately 50μm. Position the distance sensor above the gasket to track the axial displacement changes of the specimen. As this sensor operates using infrared technology, ensure there are no obstructions between the sensor and the gasket.
4) Given that the uniaxial compressive strength of the backfill material is significantly lower than that of rock, gravitational loading is not utilized. Instead, a peristaltic pump is employed to inject hydraulic oil, maintaining load stability. Operate the hand pump to ensure the specimen makes complete contact with the apparatus until the pressure gauge indicates an initial reading. Subsequently, close the hand pump valve, open the peristaltic pump valve, and use the latter to incrementally increase the load to the desired level. Upon reaching the target load, simultaneously activate the distance sensor and static strain gauge to record the experimental data.
5) Maintain a constant load for 24 hours before progressively increasing the peristaltic pump pressure to the subsequent load level. Continue this process until the loading procedure concludes or the specimen fails.
6) Open the hydraulic valve to relieve the pressure and carefully remove the specimen.
7) In order to avoid the potential impact of differences between specimens on test results, the number of tests should be carried out at least 3 times. In the analysis process, the representative results are taken for analysis.

**Fig 2:**
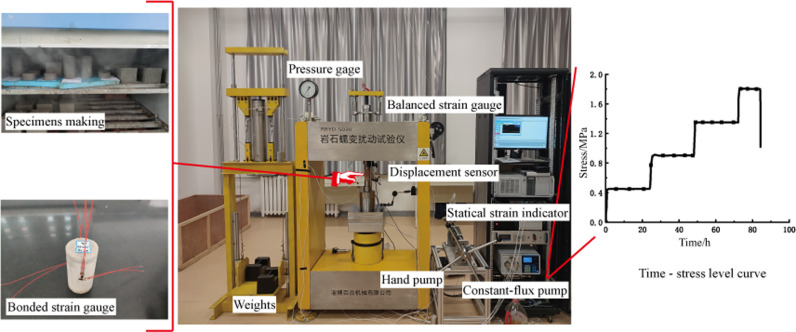
The process of creep experiment.

The design of creep graded loading stresses according to the results of uniaxial compression test is shown in Table 1 creep stage loading stress level, and the test flow is shown in Fig 2.

**Table 1:** creep stage loading stress level.

Test NO.	Creep stage loading stress level/MPa			
	1st stage 20% $R_{c}$	2nd stage 40% $R_{c}$	3rd stage 60% $R_{c}$	4th stage 80% $R_{c}$
1	0.46	0.92	1.38	1.84
2	0.45	0.91	1.30	1.82
3	0.46	0.93	1.40	1.86
4	0.44	0.90	1.30	1.79
5	0.45	0.90	1.35	1.80

**Fig 3:**
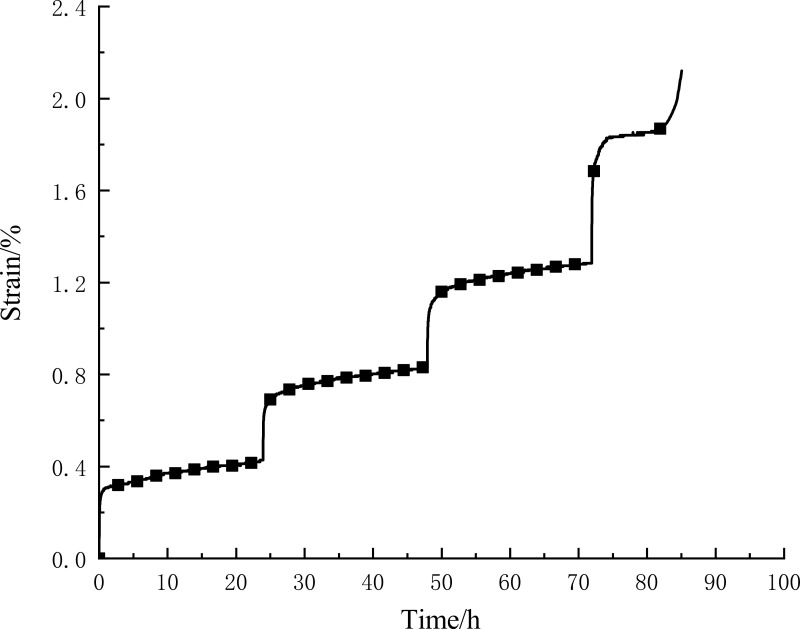
Creep curve of the CTB.

The time-strain curve during the creep test is shown in Fig 3, and the total strain during the creep of the CTB is 2.120%. The strain of the CTB is divided into instantaneous strain and creep strain, in which the instantaneous strain increment of each level of loading is 0.2520%, 0.2640%, 0.2680%, 0.3840%, accounting for 55.10% of the total strain, and creep damage is produced during the fourth level of loading, in which the strain increase is counted in the creep strain, and the creep strain accounts for 44.90% of the total strain. It indicates that the main strain in the creep process is the instantaneous strain.

## 3. Characteristics of creep hardening-damage of the CTB

### 3.1. Characteristics of creep damage of the CTB

The CTB is a composite cementitious material made of tailing sand, cement mixed with water and auxiliary additive materials, which is a porous medium with poor densification, wide distribution of cracks and pores, etc. In the early stage of applying stress, the pores and cracks inside the CTB will be compacted firstly to produce irreversible plastic changes, and there is a relative hardening of the strength, which is mainly manifested as a transient compression and densification process at this stage, and the internal damages are still very small and There is no obvious change characteristics.

**Fig 4:**
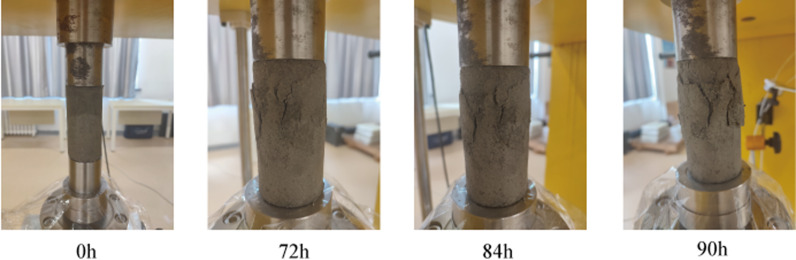
Creep failure process of CTB.

With the increase of stress level and the development of creep time, the CTB is deformed due to long-term compression, under the loading of stress, the non-homogeneity of the CTB makes the internal microscopic particles slip and deformation, resulting in a certain degree of plastic deformation, and the pore around the particles of the inter-particle misalignment occurs, resulting in small cracks and gradually expanding, expansion, and obvious damage. CTB in the hardening effect and damage effect of the joint action, manifested as a long time creep deformation, the surface of the CTB continues to produce new cracks, expanding, extending, with the passage of time, the ability of the CTB to resist deformation continues to weaken, the damage effect is more and more intense, when the damage accumulates to a certain extent, the creep rate increases suddenly, crack width intensified, the CTB lateral strain increases expansion, and eventually Eventually, due to the deformation development to crack through the loss of bearing capacity, manifested as “Y” type damage (Fig 4).

### 3.2. Creep-hardening-damage characterization of the CTB

**Fig 5:**
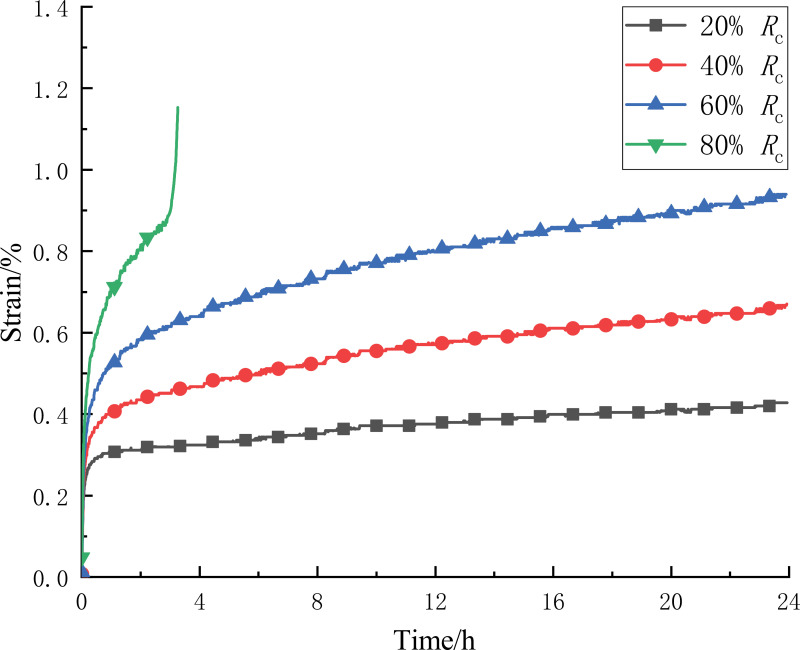
Creep curve treated by Chen loading method.

Chen’s loading method was used to process the data, and the creep curve of the CTB was obtained as in Fig 5. This method takes into account the effect of rheological medium on the memory of loading history, and the creep curve obtained by processing the creep results under fractional loading is closer to the real creep results under separate loading [24]. In this way, creep curves under multiple different loads can be obtained on a single specimen. Compared with separate loading, it can not only save the test cost, but also shorten the test period greatly. During the creep loading process, the CTB specimen had three stages of decelerated creep, isokinetic creep and accelerated creep. With the increase of stress level, the time of deceleration creep is also gradually extended, the first three levels of stress level deceleration creep stage time were 1.006h, 1.528h, 2.233h, and then enter the isokinetic creep stage. In the fourth level of stress level deceleration creep stage time is 2.894h, at this time the CTB specimen subjected to the stress exceeds its long-term strength, and then quickly enter the accelerated creep stage, the specimen occurred damage

The creep rate of the CTB in the first three isokinetic creep stages is 0.5016%/h, 1.047%/h, 1.417/h. With the increase of stress level, the creep rate in the isokinetic creep stage gradually increases, and the deformation capacity of the CTB is also increasing. At the beginning of each stress level, the pores and cracks inside the CTB were instantly compacted, and the creep rate decreased abruptly, reflecting the instantaneous hardening characteristics of the CTB. Under the first three levels of stress, creep hardening and damage work together, the creep rate continues to decrease with the development of time, indicating that the creep hardening feature exists throughout the decelerated and stable creep process, and is particularly significant in the decelerated creep stage, and at the same time, with the development of creep damage continues to accumulate, the decelerated creep experience time increases with the increase in stress level, indicating that the decelerated creep stage of damage accumulation of the CTB with the increase in stress level, and at any one time, the damage accumulation is higher than that at the beginning of each stress level. This indicates that the damage accumulation in the stage of decelerated creep of the CTB increases with the stress level, and the damage effect in the stage of decelerated creep is always stronger than that in the stage of stabilization at any stress level. Under the loading of the fourth stress level, the CTB did not undergo isotropic creep, but produced accelerated creep, the deformation increased rapidly, and eventually the internal cracks developed and could not be self-supporting, resulting in creep damage. The hardening characteristics are only manifested in the creep rate before the sudden increase, in the accelerated creep stage, the damage characteristics are significant, the damage accumulates to the peak value, and the specimen destabilizes and destroys.

**Fig 6:**
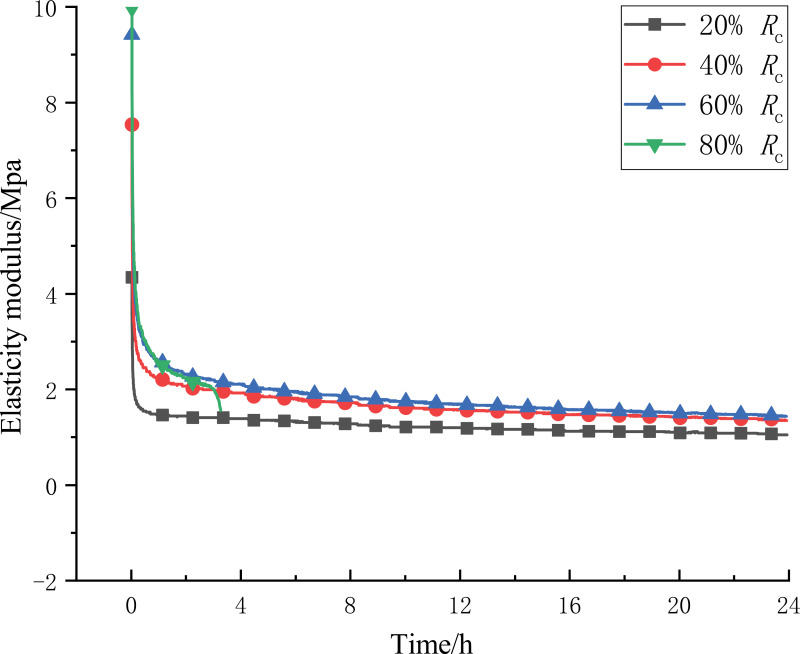
Elastic modulus curve of the CTB.

As an important mechanical index to measure the deformation process of the specimen under load, the modulus of elasticity can reflect the strain hardening or strain softening of the specimen, based on which, according to the results of the creep test, the change of the modulus of elasticity of the 1:6 CTB at all levels of stress is shown in Fig 6. The initial instantaneous modulus of elasticity increases with the increase of stress level, indicating that the degree of instantaneous hardening is enhanced with the stress level step by step, and the strength of the CTB is strengthened with the increase of stress; the modulus of elasticity decreases with the development of time at the first three levels of stress, and in the stage of isotropic creep, the modulus of elasticity is stabilized at 1.071MPa, 1.381MPa, and 1.468MPa, and increases with the increase of stress level. The stress level increases, indicating that the CTB with the development of creep internal damage is also produced continuously, the degree of damage with the stress level increases more intense, the interaction of the creep process in the CTB of the overall degree of hardening with the stress level increases gradually weakened.

In the whole creep process of the CTB, the hardening effect has existed since the early stage of the applied stress level, and the hardening effect is especially significant when the stress level is low, and the strength of the CTB increases. When the stress level gradually increased, the CTB particles of the misalignment between the CTB damage, macroscopic manifestation of the surface crack expansion, in the hardening and damage under the joint action of long-term stable creep deformation. With the further increase of the stress level, the hardening effect of the CTB is weakened, and the damage accumulates continuously, so that the strength of the CTB undergoes the change process of firstly increasing and then weakening, and eventually the damage accumulates too much, and the cracks penetrate through, and the CTB destabilizes and destroys.

## 4. The “K-r” creep model construction based on time-hardening theory

### 4.1. Creep modeling

Based on the aforementioned analysis, the creep process of CTB stems from the continuous progression and joint influence of the hardening and damage effects of CTB. During the deceleration and steady creep stages, the mechanical properties of the CTB are enhanced or strained, whereas in the accelerated creep stage, the mechanical properties of the CTB are weakened. The time-hardening theoretical relationship proposed by Kachanov [25] was adopted:


$$D={\left[\frac{\sigma }{K\left(1-D\right)}\right]}^{r}$$ (1)

Where, *D* is the damage variable, *σ* is the constant stress of each stage in the creep test of the CTB, *K* and *r* are the material constants of the CTB.

Take t:0~t*, D* :0~D to integrate the above formula, and obtain the creep damage equation:


$$D=1-{\left(1-\frac{t}{t_{f}}\right)}^{\frac{1}{r+1}}$$ (2)

Where, *t* is creep time, $t_{f}$ is creep life.

When D = 1, then:


$$t_{f}=\frac{1}{r+1}{\left(\frac{\sigma }{K}\right)}^{-r}$$ (3)

Under uniaxial compression stress $\sigma_{0}$, the CTB specimen has an initial height of $H_{0}$ and an initial cross section area of $F_{0}$ before loading. When t =0, the specimen generates an instantaneous elastic strain $\varepsilon_{0}$, and the height of the specimen is compressed to $H_{0}^{\text{'}}$.


$$H_{0}^{'}=H_{0}（1-\varepsilon_{0}）$$ (4)

If the creep strain of the specimen at time t is *ε*^′^ and the height is H, then the total strain ε at time *t* is the result of the combined action of the instantaneous elastic strain and the creep strain.


$$\text{ε}=\frac{H_{0}\varepsilon_{0}+H_{0}^{'}\varepsilon^{\prime }}{H_{0}}=\varepsilon_{0}+\left(1-\varepsilon_{0}\right)\varepsilon^{\prime }$$ (5)

$$\text{ε}=\frac{H_{0}-\text{H}}{H_{0}}=1-\frac{\text{H}}{H_{0}}$$ (6)

At the constant stress level lower than the uniaxial compressive strength, the CTB specimen will have internal damage, resulting in creep strain and creep failure of the CTB. The damage theory can well describe the damage process of the specimen. The instantaneous section area of the CTB specimen at time t is $F_{0}^{\text{'}}$. Accordingly, the instantaneous mean stress at time *t* is σ. In the creep process, the axial load acting on the CTB specimen is constant, which can be calculated by the product of stress and section area:


$$\sigma_{0}F_{0}=\text{σ}F_{0}^{'}$$ (7)

According to rock damage theory, damage degree is represented by damage variable *D*,


$$\text{σ}=\left(1-D\right)\sigma^{\prime }$$ (8)

$$\sigma^{\prime }=E_{0}\text{ε}$$ (9)

Where, *σ*^′^ is the effective stress and *D* is the damage variable at time *t*.Then the damage variable *D* can be expressed as:


$$D=\frac{F_{0}^{'}-F}{F_{0}^{'}}=1-\frac{F}{F_{0}^{'}}$$ (10)

According to damage theory, damage is caused by holes and cracks in the specimen. According to the mass conservation principle or the volume conservation principle of the undamaged part of the damage process [26–28]:


$$F_{0}H_{0}=FH$$ (11)

Substitute equation namely, (6) into equation (11), then the undamaged section area is:


$$F=\frac{F_{0}}{1-\text{ε}}$$ (12)

Bring equation (7) and (12) into equation (10), then:


$$\text{D}=1-\frac{\sigma }{\sigma_{0}\left(1-\text{ε}\right)}$$ (13)

By putting equation (3) and (13) into equation (2), the “ K−r “creep constitutive equation can be obtained:


$$\varepsilon =1-\frac{\sigma }{\sigma_{0}{\left(1-t（r+1）（\frac{\sigma }{K}{）}^{r}\right)}^{\frac{1}{r+1}}}$$ (14)

### 4.2. Creep model validation

**Fig 7:**
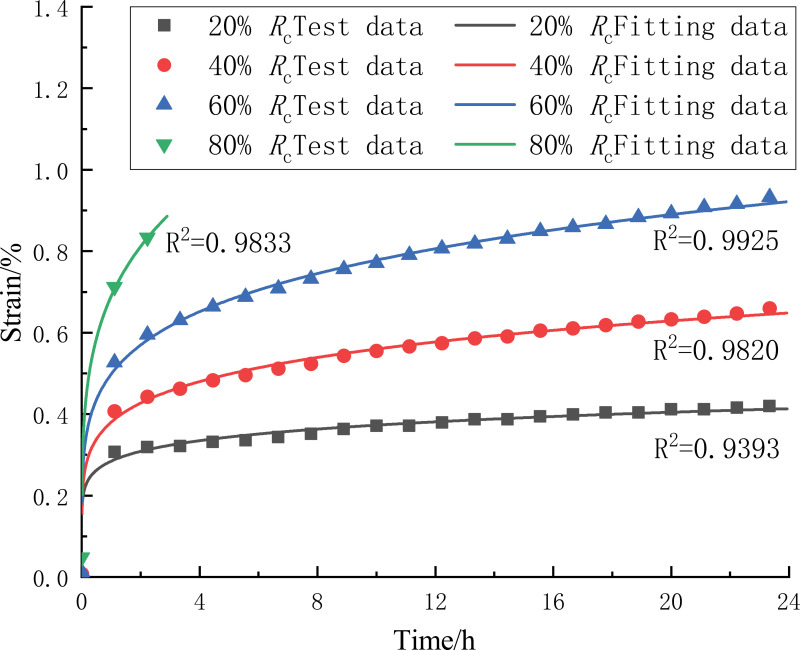
Creep fitting curve of CTB.

Based on the above process to determine the parameters in the creep model, the use of least squares fitting method of the CTB creep test data processing, will be fitted to the model creep curve and the test data for comparison and analysis (Fig 7), the R2 are above 0.93, the maximum fit reaches 0.99. It can be seen that the creep constitutive model not only accurately describes the CTB under different stress levels of the creep stage, but also more comprehensive characterization of the CTB under the action of hardening - damage creep characteristics. comprehensively characterize the creep properties of the CTB under hardening-damage effects.

The obtained creep model parameters are shown in Table 2 Model parameters of creep. With the increase of the stress level, the material constants K and r related to the CTB in the fitted creep model also increase. Combined with 1:6 the change of elastic modulus of the CTB with the stress level, the results show that, at the same stress level, the CTB has the largest elastic modulus at the loading moment, and the hardening characteristics of the CTB are obvious, and with the extension of the loading time, the CTB gradually undergoes damage, the modulus of elasticity decreases, and the creep deformation increases; at the same loading moment, the K, r of the CTB increases with the improvement of the stress level, and the CTB damage Characteristics are obvious, and the hardening capacity of the CTB increases with the increase of the stress level. According to the fitting results, the model better reflects the creep deformation characteristics of the 1:6 CTB, reflecting that the CTB is under the joint action of hardening and damage to produce creep damage.

**Table 2:** Model parameters of creep.

Stress level	K	r
20% $R_{c}$	**0.2843**	**0.1177**
40% $R_{c}$	**0.3800**	**0.1681**
60% $R_{c}$	**0.4979**	**0.1938**
80% $R_{c}$	**0.6891**	**0.2355**

We know that the properties of backfill are very different, and that the tailings after different ore separation in different regions are different. The cementing agent added is also completely different. Therefore, the creep properties of backfill are full of uncertainties, and it is difficult to have a unified model to describe them. K and r are the material constants of the CTB, if the CTB is different, K and r are different. Compared with the existing creep models of other similar materials, the influence of different creep models will not be considered here, and only K and r will be affected. The specific advantage is that the values of K and r can be obtained through basic mechanical tests and a certain amount of calculation. The creep model of CTB can be obtained by fitting analysis.

## 5. Discussion

The creep intrinsic model established above can better respond to the creep deformation of 1:6 CTB, in order to determine the influence of relevant parameters on the creep model, take the creep curve at 60% $R_{c}$ stress level as an example, change the value of different parameters, and analyze the influence of parameter changes on the model.

### 5.1. Effect of K

**Fig 8:**
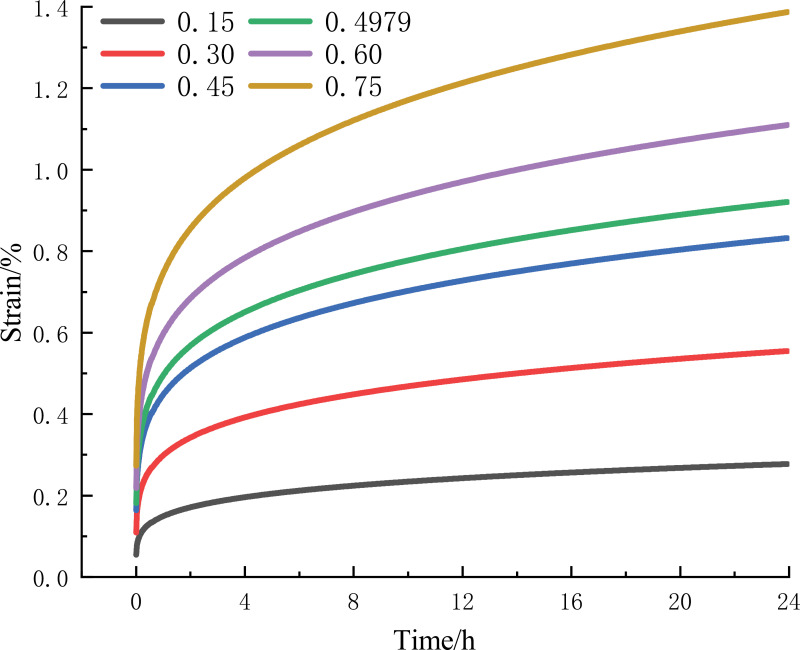
60% $R_{c}$ creep curves corresponding to different K values.

As shown in Fig 8, for the creep curve at 60% $R_{c}$ stress level, keep the stress and other parameters unchanged, change the different values of K. As the value of K increases, the deceleration creep time of the creep curve increases gradually. And then isokinetic creep stage of the deformation is proportional to the increase in the creep rate is basically the same, indicating that the K value affects the deceleration creep time.

### 5.2. Effect of r

**Fig 9:**
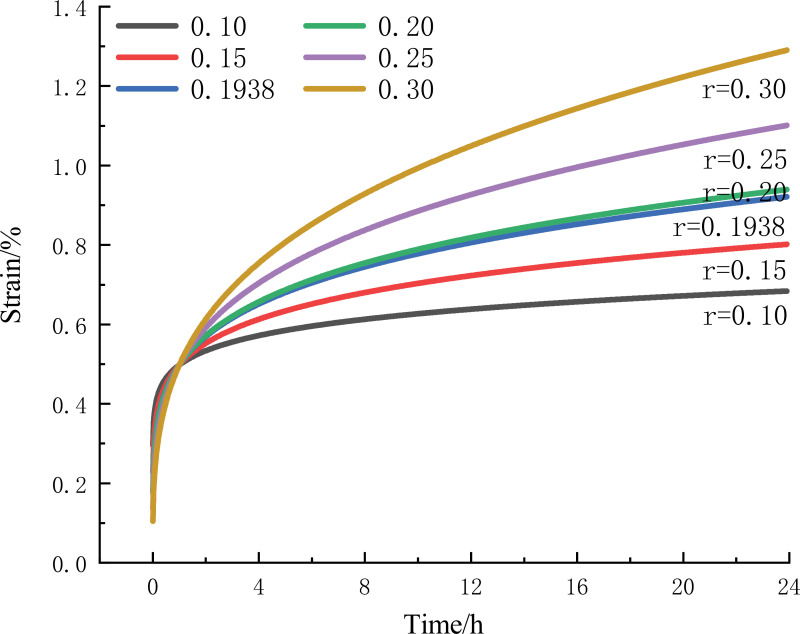
60% $R_{c}$ creep curves corresponding to different r values.

As shown in Fig 9, for the creep curve at 60% $R_{c}$ stress level, keeping the stress and other parameters unchanged and changing the different values of r, the slope of the creep curve increases as the r value increases, and the creep rate increases in the isokinetic creep stage. Different r value conditions of deceleration creep time in the same time period, indicating that the r value affects the creep rate of isokinetic creep.

From the above discussion, despite the small sample size, the results are alignment with existing basic creep theories. The parameter K affects the deceleration creep time of the deceleration creep stage, and the parameter r affects the creep rate of isokinetic creep. It can predict the long-term creep behavior of CTB quickly and judge the stability and safety of mine filling engineering. In the actual work process, by adjusting the material ratio of the CTB and changing the k and r values of the CTB, the performance of the CTB is more consistent with the actual work of the mine, which provides decision support for the stability and safety of mine filling engineering.

## 6. Conclusion

(1) In the stage of instantaneous deformation and decelerated creep, creep hardening is the most obvious characteristic of the CTB with cement-tailings ratios of 1:6; in the stage of long-term stable creep, creep hardening and damage work together to maintain equilibrium; in the stage of accelerated creep, the damage characteristic is significant.
(2) Under the joint action of creep hardening and damage, the internal cracks of the CTB are constantly compacted, and with the increase of stress, the internal particles produce slip and deformation, and cracks are produced on the surface of the specimen, and the strength of the CTB undergoes the process of strengthening and then weakening, and finally “Y” type damage occurs due to the accumulation of damage.
(3) According to the creep hardening-damage mechanism of the CTB under stress loading, the time hardening function and damage variables are introduced to construct an isomorphic model in line with the nonlinear process of the creep of its 1:6 CTB, which is verified to be in good agreement with the experimental data after fitting, and it characterizes the creep mechanical properties of the CTB under the action of hardening-damage very well. By analyzing the values of different parameters, the parameter K affects the deceleration creep time of the deceleration creep stage, and the parameter r affects the creep rate of isokinetic creep.
(4) The creep characteristics of CTB directly affect the long-term stability and safety of mine engineering. In order to better predict and evaluate the long-term behavior of mine structure, according to the creep characteristics of CTB affected by K and r values, the creep characteristics of CTB can be improved and its long-term service performance can be improved by adjusting the material ratio of CTB. In the future, the creep characteristics of CTB under the influence of environmental factors will be further investigated to understand the long-term behavior of CTB more comprehensively. It is helpful to evaluate the long-term impact of mine environment on CTB performance.

## Supporting information

S1 Data The values used to build figures. (XLSX)

## References

[1] Guo Y, Ran H, Feng G, Du X, Zhao Y, Xie W. Deformation and instability properties of cemented gangue backfill column under step-by-step load in constructional backfill mining. Environ Sci Pollut Res. 2021;29(2):2325–41. doi: 10.1007/s11356-021-15638-z 34370192

[2] Feng Y, Sun G, Liang X, Liu C, Wang Y. Experimental study on the evolution law of mesofissure in full tailing cemented backfill. Adv Material Sci Eng. 2020;2020(1). doi: 10.1155/2020/8845285

[3] Ran H, Guo Y, Feng G, Qi T, Du X. Creep properties and resistivity-ultrasonic-AE responses of cemented gangue backfill column under high-stress area. Int J Min Sci Technol. 2021;31(3):401–12. doi: 10.1016/j.ijmst.2021.01.008

[4] Wu P, Chen B, Liang B, Sun W, Jin J, Lv Z, et al. Research on the bearing creep characteristics and constitutive model of gangue filling body. Sci Rep. 2024;14(1):15207. doi: 10.1038/s41598-024-66271-y 38956294 PMC11220082

[5] Qiu J-P, Yang L, Xing J, Sun X-G. analytical solution for determining the required strength of mine backfill based on its damage constitutive model. Soil Mech Found Eng. 2018;54(6):371–6. doi: 10.1007/s11204-018-9483-7

[6] Li M, Zhang J, Meng G, Gao Y, Li A. Testing and modelling creep compression of waste rocks for backfill with different lithologies. Int J Rock Mech Min Sci. 2020;125:104170. doi: 10.1016/j.ijrmms.2019.104170

[7] Yin S, Shao Y, Wu A, Wang Z, Yang L. Assessment of expansion and strength properties of sulfidic cemented paste backfill cored from deep underground stopes. Const Build Mater. 2020;230:116983. doi: 10.1016/j.conbuildmat.2019.116983

[8] Tu B, Liu L, Cheng K, Zhang B, Zhao Y, Yang Q, et al. A constitutive model for cemented tailings backfill under uniaxial compression. Front Phys. 2020;8:9. doi: 10.3389/fphy.2020.00173

[9] Feng G, Ran H, Guo J, Guo Y, Li C. Experimental investigation on the deformation and strength properties of cemented gangue backfill column under long-term axial compression. Structures. 2022;43:1558–72. doi: 10.1016/j.istruc.2022.07.068

[10] Wang J, Yang S, Qi Y, Cong Y. Study on Bingham fractional damage model of backfill material under different moisture content conditions. PLoS One. 2024;19(1):e0295254. doi: 10.1371/journal.pone.0295254 38241329 PMC10798546

[11] Qi L, Wang J, Yan J, Jiang W, Ge W, Fang X, et al. Engineered extracellular vesicles with sequential cell recruitment and osteogenic functions to effectively promote senescent bone repair. J Nanobiotechnol. 2025;23(1):107. doi: 10.1186/s12951-025-03168-6 39939879 PMC11823168

[12] Hamami M. Experimental and numerical studies of rock salt strain hardening. Geotech Geol Eng. 2006;24(5):1271–92. doi: 10.1007/s10706-005-1880-9

[13] Cao W-G, Zhao H, Li X, Zhang Y-J. Statistical damage model with strain softening and hardening for rocks under the influence of voids and volume changes. Can Geotech J. 2010;47(8):857–71. doi: 10.1139/t09-148

[14] Cao W, Chen K, Tan X, Chen H. A novel damage-based creep model considering the complete creep process and multiple stress levels. Comput Geotech. 2020;124:103599. doi: 10.1016/j.compgeo.2020.103599

[15] Cai T, Feng Z, Jiang Y. An improved hardening-damage creep model of lean coal: a theoretical and experimental study. Arab J Geosci. 2018;11(20):12. doi: 10.1007/s12517-018-4012-6

[16] Cheng A, Zhou C, Huang S, Zhang Y, Pei M. Study on the nonlinear deformation characteristics and constitutive model of cemented tailings backfill considering compaction hardening and strain softening. J Mater Res Technol. 2022;19 4627–44. doi: 10.1016/j.jmrt.2022.06.174

[17] Liu C, Zhu L, Zhao M, He Z, Ding Z, Wang Y, et al. Experimental study on creep characteristics of cemented coal gangue backfill under uniaxial compression. Int J Coal Prep Util. 2024 1–22. doi: 10.1080/19392699.2024.2424764

[18] Guo L, Chen Q, Wu Y, Zhang Q, Qi C. Numerical simulation of adjacent stope interaction and parametric analysis of the creep behavior of rock mass. J Mater Res Technol. 2022;19:2063–76. doi: 10.1016/j.jmrt.2022.05.112

[19] Qi C, Fourie A. Numerical investigation of the stress distribution in backfilled stopes considering creep behaviour of rock mass. Rock Mech Rock Eng. 2019;52(9):3353–71. doi: 10.1007/s00603-019-01781-0

[20] Wang Z, Yang P, Lyu W, Yu G, Yang C. Study of the backfill confined consolidation law and creep constitutive model under high stress. Geotech Testing J. 2018;41(2):390–402. doi: 10.1520/gtj20160263

[21] Xu B, Li Y, Li J, Luo L, Lu B. Nonlinear stress growth and failure characteristics of gangue-cemented backfill. Constr Build Mater. 2024;424:135938. doi: 10.1016/j.conbuildmat.2024.135938

[22] Zhang J, Deng H, Duan G, Wan L, Luo Z, Sun X. Experimental study on the creep characteristics of cemented backfill in a goaf under water pressure. Adv Mater Sci Eng. 2020;2020(1). doi: 10.1155/2020/3815397

[23] LI N, CHEN C, ZHU S, MAO F. Research on creep characteristics and creep model of red clay considering effect of dry density. J Cent South Univ Sci Technol). 2020;51(08):2174–82. doi: 10.11817/j.issn.1672-7207.2020.08.013

[24] Tan T-K, Kang W-F. Locked in stresses, creep and dilatancy of rocks, and constitutive equations. Rock Mech. 1980;13(1):5–22. doi: 10.1007/bf01257895

[25] Kachanov LM. On time to rupture in creep conditions. Izviestia Akademii Nauk SSSR, Otdelenie Tekhnicheskikh Nauk, 1958;8:26–31 (in Russian).

[26] Hoff NJ. The necking and the rupture of rods subjected to constant tensile loads. J Appl Mech. 1953;20(1):105–8. doi: 10.1115/1.4010601

[27] Hu B, Wang Z, Li J, Wei E, Ma L, Liu J, et al. A new shear creep damage model for rock masses after considering initial damage. PLoS One. 2023;18(3):e0280793. doi: 10.1371/journal.pone.0280793 36972268 PMC10042354

[28] Kovrizhnykh AM, Baryshnikov VD, Khmelinin AP. Determining time-to-failure in rocks using long-term strength criterion. J Min Sci. 2023;59(6):911–8. doi: 10.1134/s1062739123060042

